# The Fibrolytic Enzyme Profiles and the Composition of Fungal Communities in Donkey Cecum-Colon Ecosystem

**DOI:** 10.3390/ani12040412

**Published:** 2022-02-09

**Authors:** Zhenwei Zhang, Yonghui Wang, Bingjian Huang, Mingxia Zhu, Changfa Wang

**Affiliations:** Liaocheng Research Institute of Donkey High-Efficiency Breeding and Ecological Feeding, Liaocheng University, Liaocheng 252059, China; qingyibushuo@163.com (Z.Z.); wyh19920729@163.com (Y.W.); 17853148163@163.com (B.H.); zhumingxia@lcu.edu.cn (M.Z.)

**Keywords:** donkey, caecum, colon, fibrolytic enzyme, fungal composition

## Abstract

**Simple Summary:**

The donkey hindgut is a microbial-rich ecosystem in which caecum and colon fungi play an important role in dietary fiber degradation. In addition, the fibrolytic enzymes produced by hindgut microorganisms are key to the ability of equines to hydrolysis plant fiber. In the present study, the fibrolytic enzyme activities within donkey caecum and colon were firstly measured by spectrophotometry. The dorsal colon presented a higher fibrolytic enzyme activity in comparison with caecum. The fungal community composition along donkey caecum and colon was determined by sequencing an internal transcribed spacer region (ITS) using Illumina MiSeq. The predominant fungi at phylum level were *Ascomycota*, *Basidiomycota*, and *Neocallimastigomycota*. The *Aspergillus*, *Wallemia*, *Phanerochaete*, *Fusarium*, and *Penicillium* were detected as the dominant genera, but their metabolic and functional significance in donkey cecum-colon ecosystem need further investigation. In terms of the anaerobic fungi *Neocallimastigomycota*, its abundance was greater in donkey colon than in caecum. The relative abundance of enzymes related to plant cell wall breakdown were also predicted by PICRUSt, and they were also greater in donkey colon than in caecum. The present study provided new information about fibrolytic enzyme profiles and fungal communities in donkey hindgut. The findings could therefore contribute to the further understanding of the fungal taxa and their dietary fiber degradation mechanisms in donkey hindgut ecosystem.

**Abstract:**

The fibrolytic enzymes and the hindgut fungi in donkey cecum-colon ecosystem play an important role in dietary fiber digestion. A better understanding of the fibrolytic enzyme profiles and the fungal community along donkey caecum and colon is key for optimizing hindgut function. In the present study, the fibrolytic enzyme activities within donkey caecum and colon were firstly measured by spectrophotometry. Activities of carboxymethyl cellulase, avicelase, xylanase, and acetyl esterase were greater in donkey dorsal colon than in caecum, indicating that the colon microorganisms may be more efficient in producing fibrolytic enzymes compared to caecum microbes. The fungal community composition along donkey hindgut was determined by sequencing ITS region using Illumina MiSeq. Three fungal phyla were identified by sequence comparison: *Ascomycota* (66.8%–74.4%), *Basidiomycota* (21.6%–30.9%), and *Neocallimastigomycota* (0.9%–3.3%). The *Aspergillus*, *Wallemia*, *Phanerochaete*, *Fusarium*, and *Penicillium* were detected as the dominant genera, but their metabolic and functional significance in donkey cecum-colon ecosystem need further investigation. In terms of the anaerobic fungi *Neocallimastigomycota*, its abundance was greater in donkey colon than in caecum (*p* < 0.05), indicating that the donkey hindgut region was associated with differences in fungal community composition. Moreover, the relative abundance of enzymes related to plant cell wall degradation were predicted by PICRUSt, and they were also lower in caecum than in colon. The present study provided new information about fibrolytic enzyme profiles and fungal composition in donkey hindgut ecosystem.

## 1. Introduction

Donkeys have evolved as free-grazing herbivores with a specialized and enlarged hindgut. The equine hindgut is mainly comprised of two fermentative compartments, the caecum and colon, which together account for two-thirds of the volume of donkey’s gastrointestinal tract [1]. Microbial fermentation of plant structural polysaccharides in donkey hindgut results in the production of volatile fatty acids (VFAs), which are a major source of energy for the host [2].

Among hindgut microorganisms, both the bacteria and the fungi play a pivotal role in the hindgut fermentation and the degradation of feedstuffs [2]. Nowadays, increasing studies have been successfully performed to reveal the changes and functions of the hindgut bacteria community in equines [3,4]. However, little information is available regarding the composition of fungal communities in the equine hindgut ecosystem. The current knowledge of gut fungi has focused on the strictly anaerobic fungi (phylum *Neocallimastigomycota*) and is mostly derived from ruminant based studies [5,6].

Based on physiological and developmental characteristics, *Neocallimastigomycota* is generally divided into the monocentric rhizoidal, the polycentric rhizoidal, and the bulbous genera [5]. Anaerobic fungi were reported to be present in domesticated and non-domesticated equine species, and their diversity in donkeys was shown to be higher when compared to that of ponies and pony × donkey hybrids [2]. Recently, Edwards et al. determined the fecal community composition of anaerobic fungi in donkeys, and the genus *Caecomyces*, SK3, KF1, and *Piromyces* were found in the fecal samples [2]. The presence of these anaerobic fungi may enable the donkeys to utilize fibrous feeds efficiently and persistently. However, recent studies in ruminant animals have shown that *Ascomycota* and *Basidiomycota* phyla also play an important role in the rumen digestion of cashmere goats and Holstein cows [7,8].

The fungal ribosomal RNA (rRNA) operon-based analysis has facilitated the understanding of the diversity and phylogenetic relationships of gut fungi in natural ecosystems [9]. The internal transcribed spacer (ITS) region is usually recommended as the DNA barcode marker for gut fungi [10,11]. In addition, the 28S ribosomal large subunit (28S rRNA gene (LSU); D1/D2 region) might render higher coverage of early diverging lineages of fungi, such as *Neocallimastigomycota*, *Chytridiomycota*, and *Mucoromycota* [11]. However, there is still a big challenge of how to relate LSU taxonomic sequences to the clades that until now only contained “unculturable representatives” derived ITS1 sequences [5,12]. Therefore, the ITS region remains the most accepted phylogenetic marker to assess the diversity and community structure of gut fungi.

Due to ethical noninvasive sampling, fecal samples are commonly used for fungi investigation in equines [2,7]. The understanding of equine fungi diversity and community structure within different hindgut regions is limited. It is very necessary to evaluate the diversity and composition of hindgut fungi in the anatomically specialized hindgut of donkeys. Moreover, both the bacteria and the fungi produce a broad array of fibrolytic enzymes that can facilitate the plant fragments degradation by breaking the linkages between lignin and hemicelluloses [13,14]. The digestibility of plant fiber in donkeys may be to a large extent dependent on the strong fibrolytic activity of the main-chain or the side-chain degrading enzymes, including carboxymethyl cellulase, xylanase, ferulic acid esterase, and acetyl esterase. However, neither the main-chain degrading enzymes nor the side-chain degrading enzymes have been reported in the donkey caecum and colon.

Therefore, the fibrolytic enzyme profiles were firstly determined to assess functional differences in digesting plant cell wall within the different hindgut regions in donkeys. Furthermore, the primary objective of the present study is to comparatively analyze the fungal community composition along the hindgut segments (caecum and colon) of donkeys using Illumina MiSeq sequencing by covering the ITS region.

## 2. Materials and Methods

### 2.1. Ethics Statement

The animal care and sample procedures were approved by the Institutional Animal Care Committee at Liaocheng University (Permit No. DFG21010103-1).

### 2.2. Animals and Diets

Eight jackasses (weighing 303 ± 18 kg, 2.5 years of age) belonging to the Dezhou donkey breed were enrolled. They were reared in a large-scale donkey farm in northern China and housed in stalls. Donkeys were fed twice daily at 07:00 and 19:00 h. Diet was based on corn straw ad libitum along with a commercial concentrate feed, according to the nutrient requirements reported by the NRC recommendations [15]. Donkeys had free access to water at all times. In addition, donkeys were healthy, with no history of any intestinal disorders.

### 2.3. Sample Collection

Donkeys were euthanized for non-research reasons in a local abattoir. After fasting for 12 h, they were knocked unconscious using electronarcosis of 220 V for 20 s, and then slaughtered by exsanguination using conventional humane procedures. After exteriorization of the gastrointestinal tract, the caecum, ventral colon, and dorsal colon were tied off to avoid mixing between adjacent segments. The hindgut contents from one section were collected immediately with press filtration through four layers of cheesecloth and mixed thoroughly to obtain the representative caecum, ventral, and dorsal colon sample (liquid and small particles). An aliquot of each sample (2 mL) was transferred to the labelled sterile tube and transported on wet ice back to the laboratory where they were stored at −70 °C until DNA extraction. A second aliquot (10 mL) of each sample was transferred to polypropylene tube and centrifuged at 1000× *g* at 4 °C for 15 min, and the supernatant was immediately analyzed for fibrolytic enzyme profile analysis.

### 2.4. Fibrolytic Enzyme Profile Measurement

The activity of cellulolytic enzyme including carboxymethyl cellulose (CMCase), avicelase (AVI), xylanase (XYL), feruloyl esterase (FAE), and acetyl esterase (AE) were measured by the spectrophotometer (Bio-rad, Hercules, CA, USA), as described previously [16]. For FAE activity determination, the primary centrifugal supernatants from caecum, ventral colon, and dorsal colon fluids were incubated with 2 vol of 100 μM methyl ferulate in 100 mM 3-(N-morpholino) propane sulfonic buffer at 39 °C for 30 min, and the absorbance was detected by spectrophotometer at 340 nm. Enzyme activity was calculated according to standard curves of feruloyl acid (FA) and methyl ferulate (MA):

Activity (U) = [(OD_0_ − OD_30_) − (OD_b0_ − OD_b_)] × V_r_ × Dilution/[(εMA × *l* − εFA × *l*) × V_s_]

where OD_0_ and OD_30_ is the absorbance of reaction system at 0 and 30 min, respectively; OD_b0_ and OD_b30_ is the absorbance of blank system at 0 and 30 min, respectively; V_r_ and V_s_. is the volume of reaction system and sample, respectively; εMA is the extinction coefficient of methyl ferulate; εFA is the extinction coefficient of feruloyl acid; and *l* is the pathlength.

The activities of AE, CMCase, AVI, and XYL were measured colorimetrically by using p-nitrophenyl acetate, carboxymethyl cellulose, avicael, and xylan as the substrate, respectively. The production of *p*-nitrophenol was determined at 415 nm, and the production of reducing sugars was quantified colorimetrically at 540 nm by the dinitrosalicylic acid method [17]. One unit of enzyme activity was defined as the amount of enzyme releasing 1.0 μmol of corresponding reducing products (feruloyl acid or reducing sugar or *p*-nitrophenol)/min/mL under the aforementioned conditions.

### 2.5. The DNA Extraction and PCR Amplification

Following the instructions, genomic DNA was extracted from caecum and colon fluids samples using the E.Z.N.A.^®^ soil DNA Kit (Omega Bio-tek, Norcross, GA, USA). After the evaluation of quality and quantity of extracted DNA, four samples from each caecum, ventral colon, and dorsal colon were selected and delivered to Majorbio Company (Shanghai, China) for PCR amplification and MiSeq sequencing.

For anaerobic fungal community composition analysis, barcoded amplicons comprising the ITS1–ITS2 were generated with primer pairs ITS1F (5′-CTTGGTCATTTAGAGGAAGTAA-3′) [18] and ITS2R (5′-GCTGCGTTCTTCATCGATGC-3′) [19] by an ABI GeneAmp^®^ 9700 PCR thermocycler (ABI, Arlington, VA, USA). The PCR amplification of ITS1~ITS2 gene was performed in a total volume of 20 μL (Table 1). The PCR were carried out in triplicate and using the appropriative thermal cycling conditions. The PCR product (350 bp) was excised from 2% agarose gel and purified by the AxyPrep DNA Gel Extraction Kit (Axygen Biosciences, Union City, CA, USA). The DNA concentrations of PCR products were measured using Quantus™ Fluorome-ter (Promega, Fitchburg, WI, USA) and amplicons from each reaction mixture were pooled at equimolar ratios based on the concentration of each amplicon.

### 2.6. The Illumina MiSeq Sequencing

Following the standard protocols of Majorbio Bio-Pharm Technology Co. Ltd. (Shanghai, China), the purified PCR amplicons were pooled in equimolar and paired-end sequenced (2 × 300) on an Illumina MiSeq platform (Illumina, San Diego, CA, USA). The raw reads were deposited into the NCBI Sequence Read Archive (SRA) database (PRJNA7797744).

### 2.7. Processing of Anaerobic Fungal Sequence Data

The fungal sequence data was then quality-filtered using Trimmomatic and merged by FLASH v.1.2.11 with the standard protocols described by Wang et al. [20]. After the removal of chimeric sequences, operational taxonomic units (OTUs) with 97% similarity cutoff were clustered by UPARSE (version 7.1). Venn diagrams were built using the R language (version 3.3.1). The taxonomy of each OTU representative sequence was identified by Ribosomal Database Project (RDP v.2.11) Classifier against the unite fungi ITS database (version 8.0) using confidence threshold of 0.97. The coverage and sampling effort were estimated by the determination of Good’s coverage and rarefaction curves. Unweighted and weighted uniFrac distance-based principal coordinate analysis (PCoA), fungal diversity (Shannon and Simpson index), and richness (Sobs and Chao index) were assessed by the program-Mothur (version v.1.30.2). The composition of each sample at each taxonomic level was determined and plotted into taxonomy histograms. The relative abundance of fungal community was expressed as a percentage. Kruskal-Wallis H test was used to assess the difference in the relative abundance of fungi. Fungal enzymatic abundance was predicted using PICRUSt v.1.1.0 (Phylogenetic Investigation of Communities by Reconstruction of Unobserved States, Paris, 2012, Curtis Huttenhower) software.

### 2.8. Statistical Analysis

Univariate data (fibrolytic enzyme profiles) were analyzed using the MIXED procedure of SAS 9.4. The MIXED included the fixed effect of donkey hindgut region, and the random and repeated effect of the animal (*n* = 8) as covariate. Least square mean and standard error of means (SEM) were calculated with the LSMEAN procedure of the SAS 9.4. Differences were considered at *p* < 0.05, whereas 0.05 < *p* < 0.10 were declared to be a trend. Analyses of fungal community data and ITS predicted functional profiles were carried out using the i-Sanger platform (http://www.i-sanger.com/, accessed on 15 January 2022).

## 3. Results

### 3.1. Fibrolytic Enzyme Profiles

Apart from the FAE, the activities of CMCase, AVI, XLY, and AE in caecum and ventral colon were all lower than that in dorsal colon (Table 2, *p* < 0.05).

### 3.2. Alpha Diversity

**Figure 1:**
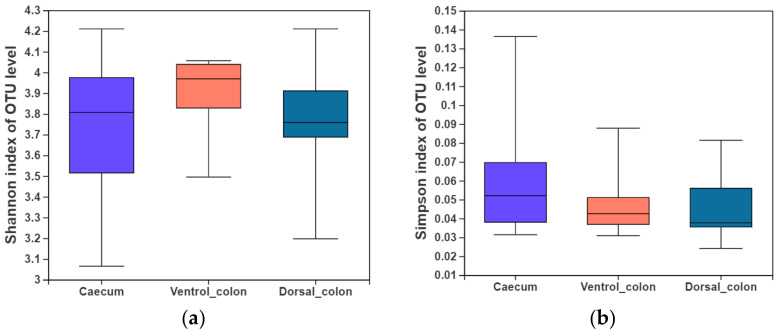

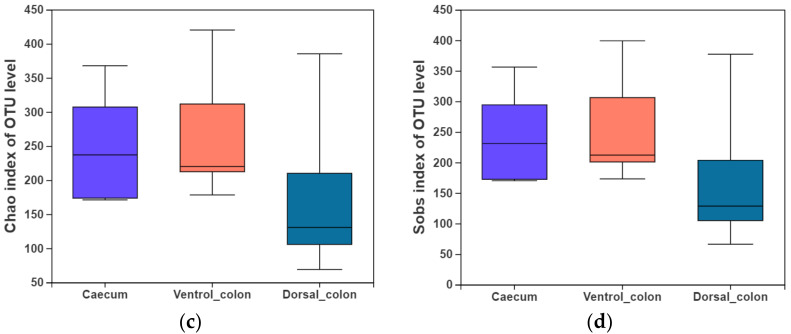
Fungal alpha diversity indices of caecum and colon in Dezhou donkeys. (**a**), Shannon index; (**b**), Simpson index; (**c**), Chao index; (**d**), Sobs index.

In the present study, indices of alpha diversity included Shannon, Simpson, Chao, and Sobs index (Figure 1). No difference occurred among donkey caecum, ventral colon, and dorsal colon (*p* > 0.05).

### 3.3. Fungal Community Composition

**Figure 2:**
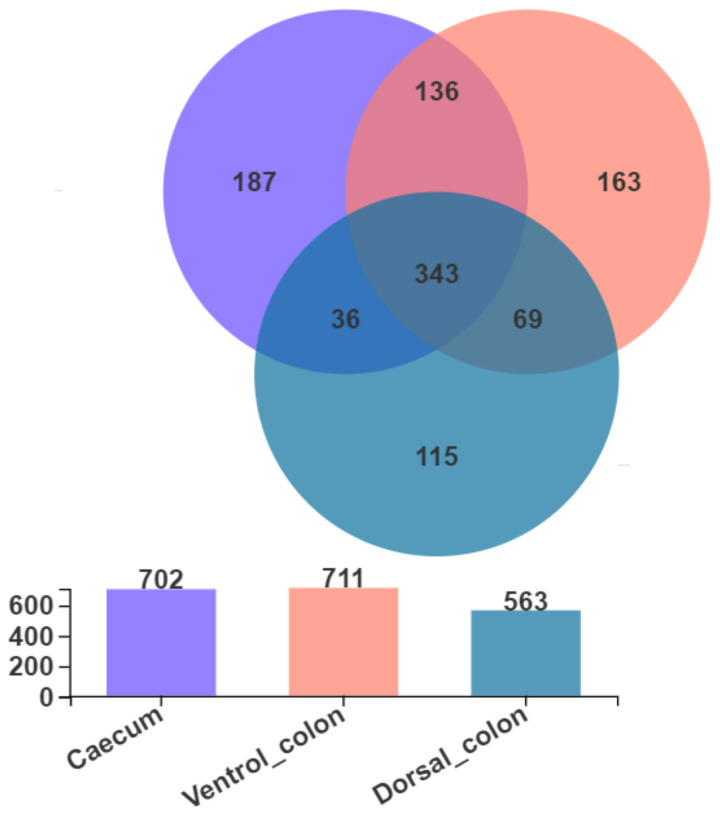
Venn diagram presented the distribution of fungal community OTUs across the caecum, ventral colon, and dorsal colon.

As shown in Figure 2, Venn diagram presented the distribution of fungal community OTUs. There were 702, 711, and 563 OTUs that were observed in donkey caecum, ventral colon, and dorsal colon, respectively. In addition, caecum shares the fungal community, including 343 OTUs, with ventral colon and dorsal colon.

**Figure 3:**
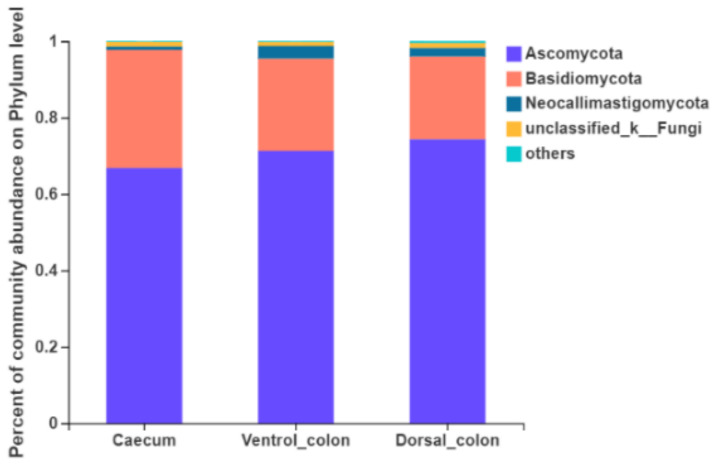
Composition of the predominant fungal phyla (relative abundance ≥1%) among donkey caecum, ventral colon, and dorsal colon (*n* = 8).

Fungi with a relative abundance of ≥1% of the total sequences in at least one of the samples were further analyzed (Figure 3). The top four predominant phylum were *Ascomycota* (66.8%–74.4% of the total sequence reads), *Basidiomycota* (21.6%–30.9%), *Neocallimastigomycota* (0.9%–3.3%) and unclassified fungi (1.0%–1.3%).

**Figure 4:**
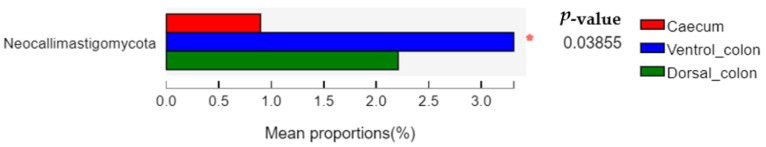
Difference in the relative abundance of fungal phyla (abundance of the phyla was expressed as %). Welch’s two-sided test was used and Welch’s inverted was 0.95. *, *p* < 0.05.

The relative abundance of *Neocallimastigomycota* in donkey caecum was significantly lower than that in the ventral and dorsal colon (Figure 4; *p* = 0.03).

**Figure 5:**
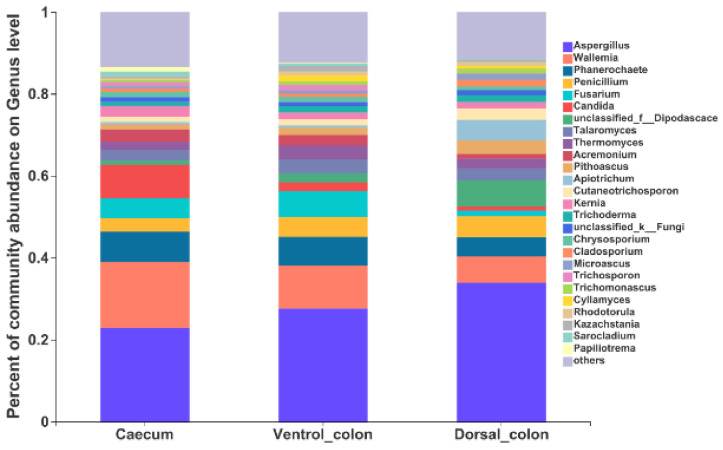
Composition of the fungal genera (relative abundance >1%) among donkey caecum, ventral colon, and dorsal colon (*n* = 8).

At the genus level, the top 10 predominant genera were *Aspergillus*, *Wallemia*, *Phanerochaete*, *Fusarium*, *Candida*, unclassified_*Dipodascace*, *Talaromyces*, *Thermomyces*, *Acremonlum*, and *Pithoascus* (Figure 5).

**Figure 6:**
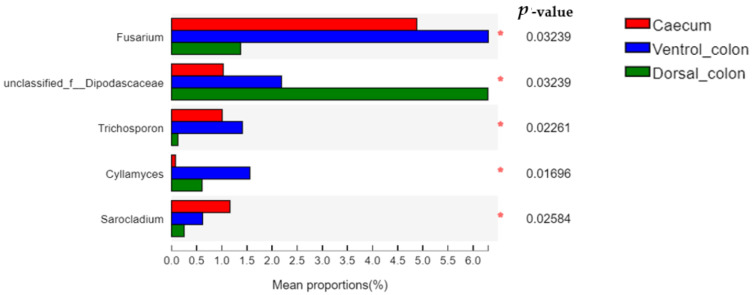
Difference in the relative abundance of fungal genera (abundance of the phyla was expressed as %). Welch’s two-sided test was used and Welch’s inverted was 0.95. *, *p* < 0.05.

The relative abundance of *Fusarium* and *Trichosporon* in donkey hindgut was ranked as: ventral colon > caecum > dorsal colon (Figure 6; *p* < 0.05). In addition, the relative abundance of unclassified *Dipodascaceae* and *Cyllamyces* in donkey colon was significantly greater than that in the caecum (*p* < 0.05).

The anaerobic fungal community belonging to the *Neocallimastigomycota* phylum were further analyzed (Table 3). The genera *Cyllamyces*, *Neocallimastix*, *Piromyces*, *Buwchfawromyces*, Unclassified_f_*Neocallimastigaceae*, and unclassified_c_*Neocallimastigomycetes* were detected within donkey hindgut. The abundance of anaerobic fungal community composition varied greatly between donkey individuals. In addition, the relative abundance of anaerobic fungi was numerically lower in the caecum than in both ventral colon and dorsal colon.

### 3.4. Beta Diversity

**Figure 7:**
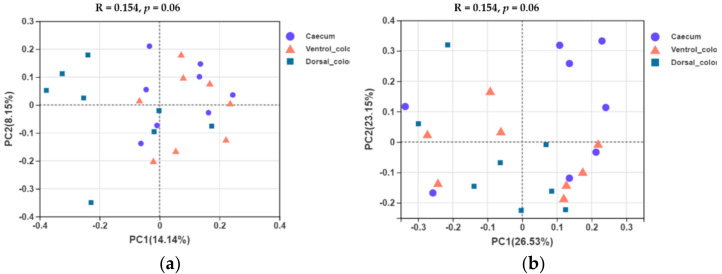
Unweighted (**a**) and weighted (**b**) unifrac based principal coordinates analysis (PCoA) of the fungal community composition of the different hindgut regions at the OTU level. Analysis used Log_10_ transformed data, and the percentage values given on each axis indicate the amount of total variation represented. PC1, first axis; PC2, second axis.

In terms of beta diversity at the OTU level, unweighted and weighted unifrac PCoA showed that there were obvious varieties between individuals for the fungal community composition. Separation of dorsal colon from caecum occurred along the first axis (PC1) of the unweighted PCoA, but no separation of caecum from ventral colon was seen (Figure 7a). No obvious separation of the samples by hindgut region was observed in the weighted PCoA (Figure 7b).

### 3.5. Prediction of Fungal Enzymatic Activity by PICRUSt (Phylogenetic Investigation of Communities by Reconstruction of Unobserved States)

**Figure 8:**
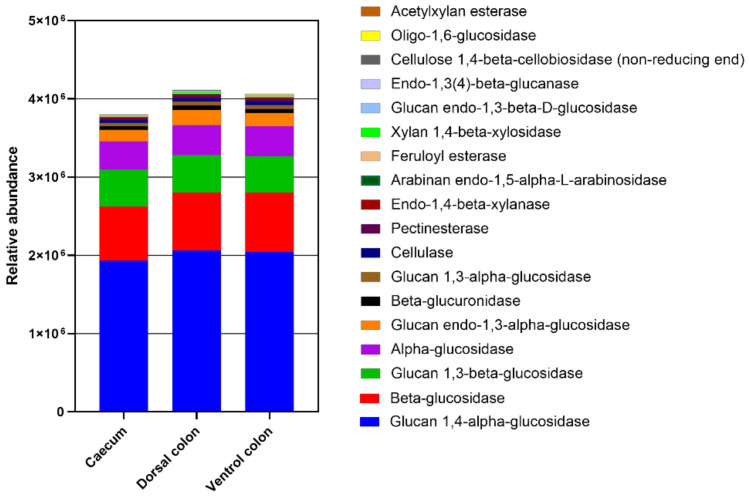
The abundance of enzymes related to plant cell wall degradation predicted by phylogenetic investigation of communities by reconstruction of unobserved states (PICRUSt). PICRUSt is a bioinformatics software package designed to predict metagenome functional content from marker gene (e.g., ITS) surveys.

The relative abundance of enzymes related to plant cell wall degradation were predicted by PICRUSt (Figure 8). These enzymes mainly included glucan 1,4-alpha-glucosidase beta-glucosidase, glucan 1,3-beta-glucosidase, alpha-glucosidase, glucan endo-1,3-alpha-glucosidase, beta-glucuronidase, glucan 1,3-alpha-glucosidase, cellulase, pectinesterase, endo-1,4-beta-xylanase, arabinan endo-1,5-alpha-L-arabinosidase, feruloyl esterase, xylan 1,4-beta-xylosidase, glucan endo-1,3-beta-D-glucosidase, endo-1,3(4)-beta-glucanase, cellulose 1,4-beta-cellobiosidase (non-reducing end), and oligo-1,6-glucosidase, acetylxylan esterase. The relative abundances of these enzymes were lower in caecum than in both ventral and dorsal colon.

## 4. Discussion

### 4.1. Fibrolytic Enzyme Profiles

The donkey hindgut is well developed and comprised of the several chambers, mainly including caecum, ventral colon, dorsal colon, and rectum [21]. In the previous study, Miyaji et al. reported that the cumulative disappearance of dietary fiber from the caecum to the rectum attained 90% of total tract digestion, indicating that the cecum and colon are the main regions for plant fiber fermentation in equines [22]. As equines themselves lack fibrolytic enzymes, the hindgut microorganisms are key to the ability of equines to hydrolyze dietary fiber [2]. Both the bacteria and the fungi can degrade dietary fiber efficiently via secreting a range of fibrolytic enzymes, such as free enzymes and cellulase multienzyme complexes [23]. Until now, there was a critical lack of knowledge regarding the fibrolytic activity among donkey hindgut. In the present study, the main-chain degrading enzymes (CMCase, AVI and XLY) and the side-chain degrading enzymes (AE) were thoroughly determined in donkey hindgut, and the activities were higher in colon in comparison with caecum. The fibrolytic activity may be related to the physical structure and chemical composition of the fibrous feeds [24]. With the incessant passage of the liquid and solid digesta in donkey hindgut, the structures of carbohydrate sources were changed and can regulate fibrolytic enzyme production. The increased lignin content and cross-linking between lignin polymers and polysaccharides in the colon digesta may promote the fibrolytic activity [25], especially the AE activity. Esterase is usually required to hydrolyze the cross-linkages in plant cell wall through phenolic compounds [4].

### 4.2. Fungal Community in Donkey Caecum and Colon

In recent years, study on the equine hindgut microbiota has increasingly improved with the advances in high throughput sequencing technology [3,4,26]. PCR amplification of universal primers for conserved regions within the rRNA genes, followed by DNA sequencing of the internal transcribed spacer (ITS), is widely used in fungal identification studies [27]. In the present study, we focused on the taxonomic affiliation of hindgut fungi by covering the ITS region with fungal wide primers (ITS1F~ITS2R) [18,19]. The results provide new information about donkey caecum and colon fungi communities. At phylum level, *Ascomycota*, *Basidiomycota*, *Neocallimastigomycota*, and unclassified fungi were the predominant fungi within donkey caecum and colon. The present result is in agreement with the result of Zhang et al. [7] and Han et al. [8] in ruminant animals. Until now, knowledge of hindgut fungi and their influence on the equine animal remains limited. Using high-throughput sequencing to study the effect of hindgut region on fungal diversity and composition in donkeys has not been described. Edwards et al. [2] determined the fungal composition of fecal samples from donkey (*Equus africanus asinus*), however, they just target the phylum *Neocallimstigomycta*. These inconsistencies on phylum level might be due to differences in primers used for sequencing. Nowadays, both the ITS region and LSU (28S rRNA gene; D1/D2) region have been recommended as the universal DNA barcode marker for fungi [11]. However, a sufficient reference database is lacking for LSU taxonomic marker genes so far [5]. It is likely that ITS will continue to be commonly used in the near future as the primary barcode to evaluate fungal diversity and community composition.

In the current study, although the Ascomycota and Basidiomycota may be the dominant fungus in donkey caecum and colon, donkey hindgut region was just associated with differences in phylum *Neocallimstigomycta*. The relative abundance of *Neocallimastigomycota* in donkey caecum was significantly lower than that in the ventral and dorsal colon. In ruminants, previous studies reported that the *Neocallimastigomycota* was the most predominant fungal phylum [28,29], and they were important in the degradation of fibrous plant materials in ruminants [7,30]. The *Neocallimastigomycota* was presumed to be an effective plant fiber degrader and is well-known for its powerful hydrolytic properties and multi-functional fibrolytic enzymes [30]. Therefore, the larger abundant of *Neocallimastigomycota* in colon digesta may enable a donkey to degrade plant fiber more effectively compared to caecum.

At genus level, *Aspergillus*, *Wallemia*, *Phanerochaete*, *Fusarium*, and *Penicillium* were the predominant genera in current study. The genus *Aspergillus* is a group of filamentous fungi with a large number of species, and they have an effective effect on plant cell wall breakdown by producing a wide range of fibrolytic enzymes [31]. The enzymes act not only on the main chain of plant cell wall polysaccharides, but also act on the substituents or the side chains like the linkages between a main-chain residue and a substituent [30]. The *Wallemia* is another xerophilic filamentous fungi with the ability to secret a series of glycosidases [32]. The glycosidase was able to degrade both α- and β- linked glycosidic bonds among di- and polysaccharides [33]. In addition, the *Phanerochaete*, *Fusarium*, and *Penicillium* were also reported to have the ability to produce various highly active enzymes, including cellulase, xylanase, and esterase [34,35,36]. From this study, the findings indicate that fungal community composition differs between different hindgut regions. The abundance of *Fusarium* and *Trichosporon* is greater in caecum than in dorsal colon. In contrast, the unclassified_*Dipodascaceae* and *Cyllamyces* in donkey colon was greater than in caecum. Nevertheless, as these genera were rarely found in herbivores, their metabolic and functional significance in donkey caecum-colon ecosystem need further investigation.

As strictly anaerobic fungi, *Neocallimastigomycota* have been most extensively studied in ruminants, but their information within equine animals is sparse. Recently, Edwards et al. observed that genus *Caecomyces* was the most predominant in the anaerobic fungal community of donkey fecal samples [2]. This is in agreement with the present study. The genus *Caecomyces*, belonging to *Neocallimastigomycota* phylum, was also detected as the dominant anaerobic fungi in donkey hindgut. In addition, the anaerobic fungi genera including *Neocallimastix*, *Piromyces*, *Buwchfawromyces*, Unclassified_f_*Neocallimastigaceae* and unclassified_c_*Neocallimastigomycetes* were also obtained from the donkey caecum and colon. The genus *Neocallimastix*, *Piromyces*, and *Buwchfawromyces* were cultured from equines [2,6]. However, Edwards et al. (2020) also observed the uncultivated genus SK3 and KF1 in donkey faeces, which was different from our findings [2]. The different donkey breeds and sample types may cause the distinct fungal compositions [37]. In addition, faecal samples did not accurately reflect the fungal community composition over the rest of the regions of the donkey hindgut [38]. For ruminant animals, Kittelmann et al. [39] noted that *Neocallimastix*, *Piromyces*, *Orpinomyces*, *BlackRhino*, *Caecomyces,* and *Cyllamyces* were the predominant anaerobic fungal genera in the rumen. This is perhaps not surprising considering the fundamental differences between hindgut herbivores and ruminants in terms of the main gut site where plant fiber degradation primarily occurs [5]. However, the genus *Neocallimastix*, *Piromyces*, and *Cyllamyces* were found to be shared in donkey hindgut and rumen. A previous study has shown that *Neocallimastix* is efficient in straw silage cell wall digestion [40], and it showed high polysaccharide hydrolase and esterase activity [41]. *Piromyces* could regulate the secretion of lignin-regulating enzymes via the fungal pathway and is capable of producing effective enzyme complex [42]. Screening of the microbial CAZyme transcripts indicated that the anaerobic fungi *Neocallimastigaceae* produced the largest share of cellulase transcripts [43]. The relative abundance of *Neocallimastix*, *Piromyces*, *Buwchfawromyces*, Unclassified_f_*Neocallimastigaceae*, and unclassified_c_*Neocallimastigomycetes* were numerically greater in colon than in caecum, which may enable the colon microorganisms to producing more fibrolytic enzymes than caecum microorganisms. Further study will be required to confirm the present observations.

### 4.3. Prediction of Fungal Enzymatic Abundance by PICRUSt

Due to the broad array of powerful plant degrading enzymes and the combined invasive growth, the fungi are key for herbivores to degrade plant cell walls [2]. There is a rare study performed on the fungal enzymes within equine animals. PICRUSt is a bioinformatics software package designed to predict metagenome functional content from marker gene (e.g., ITS). In the present study, the relative abundance of carbohydrate-active enzymes related to the degradation of cellulose, hemicellulose, pectin, and lignin were predicted. Interestingly, the relative abundance of these fibrolytic enzymes (glucan 1,4-alpha-glucosidase beta-glucosidase, glucan 1,3-beta-glucosidase, al-pha-glucosidase, glucan endo-1,3-alpha-glucosidase, beta-glucuronidase, glucan 1,3-alpha-glucosidase, cellulase, pectinesterase, endo-1,4-beta-xylanase, arabinan endo-1,5-alpha-L-arabinosidase, feruloyl esterase, xylan 1,4-beta-xylosidase, glucan endo-1,3-beta-D-glucosidase, endo-1,3(4)-beta-glucanase, cellulose 1,4-beta-cellobiosidase (non-reducing end), oligo-1,6-glucosidase, and acetylxylan esterase) were greater in colon than in caecum. Considering that the relative abundance of *Neocallimastigomycota* was significantly greater in donkey colon than in colon, the higher enzyme content for plant cell wall breakdown in donkey colon may be related to their greater *Neocallimastigomycota* abundance. This result might consequently lead to the more efficient activity in dietary fiber digestion within donkey colon, relative to caecum.

### 4.4. Welfare of the Animals

With the unique cecum-colon ecosystem, donkeys obtain nutrients and energy from high fiber diets through microbial fermentation [44]. Information regarding the basic structure of the microbial flora in donkey hindgut is of vital importance in determining which components of the microflora respond to dietary variations [44]. The anaerobic fungi community and relative enzymes reported in the present study may provide a concrete reference for understanding donkey nutritional needs and health implications. It was because the donkey hindgut inhabited a large number of anaerobic bacteria and fungi that made donkeys highly efficient at digesting poor nutritional quality fiber [45]. Forages were the basis of donkey’s diet, and the concentrated meals were discouraged from use [46]. Donkeys rarely require energy-rich concentrate, such as cereal grains, sweet feeds, or highly crushed feed [45]. Due to the high concentration of soluble carbohydrates in cereal grain diets, the feeding of such concentrates for donkey is poorly tolerated. When concentrates were given over and above the capacity of the foregut to digest them, the increased soluble carbohydrates will enter the caecum and colon [44,47]. This was implicated in the development of health problems, such as obesity, hyperlipemia, equine acidosis, and colic [45,48]. In addition, feeding more of less energy-rich feeds may also allow larger meals, giving the animals longer eating times. Therefore, healthy donkeys in practical feeding should focus on optimizing fiber utilization and providing greater proportions of highly fibrous feedstuffs, such as crop straw, grass hay, and haylage being fed as required, according to body condition and life stage [45].

## 5. Conclusions

The fibrolytic enzyme profiles within different donkey hindgut regions were firstly measured in the present study. Activities of carboxymethyl cellulase, avicelase, xylanase, and acetyl esterase were greater in donkey dorsal colon than in caecum, indicating that the colon microorganisms may be more efficient in producing fibrolytic enzymes compared to caecum microbes. The fungal community composition along donkey hindgut segments using Illumina MiSeq sequencing by covering the ITS region were determined. The predominant fungi at phylum level in donkey caecum and colon were *Ascomycota* (66.8–74.4%), *Basidiomycota* (21.6–30.9%), *Neocallimastigomycota* (0.9–3.3%) and unclassified_fungi (1.0–1.3%). The *Aspergillus*, *Wallemia*, *Phanerochaete*, *Fusarium*, and *Penicillium* were detected as the dominant genera in donkey caecum and colon, but their metabolic and functional significance in donkey caecum-colon ecosystem need further investigation. The hindgut region of donkey was associated with differences in fungal community composition. The relative abundance of *Neocallimastigomycota* in dorsal colon was greater in donkey colon than in caecum, and the genera *Cyllamyces*, *Neocallimastix*, *Piromyces*, *Buwchfawromyces*, unclassified_f_*Neocallimastigaceae*, and unclassified_c_*Neocallimastigomycetes* belong to *Neocallimastigomycota* phylum were also numerically greater in colon than in caecum. This result indicated that the high proportion of strictly anaerobic fungi in colon may enable a donkey to degrade plant fiber more effectively, compared to caecum. Moreover, the relative abundance of enzymes related to plant cell wall degradation were predicted by PICRUSt, and the relative abundances of these enzymes were also lower in caecum than in both ventral colon and dorsal colon. The findings in the current study could therefore contribute to the further understanding of the fungal taxa and their plant fiber degradation mechanisms in donkey hindgut ecosystem.

## Figures and Tables

**Table 1:** The reaction system for PCR amplification.

Items	Volume
10 × TaKaRa rTaq buffer	2 μL
2.5 mM dNTPs	2 μL
5 μM forward primer	0.8 μL
5 μM reverse primer	0.8 μL
TrTaq Polymerase	0.2 μL
template DNA	10 ng
ddH_2_O	up to 20 μL

**Table 2:** The fibrolytic enzyme profiles in cecum, ventral colon, and dorsal colon of Dezhou donkeys.

Items	Caecum	Ventral Colon	Dorsal Colon	SEM	*p*
CMCase, U	0.13 ^b^	0.10 ^b^	0.37 ^a^	0.066	0.02
AVI, U	0.14 ^b^	0.12 ^b^	0.23 ^a^	0.015	<0.01
XLY, U	2.5 ^b^	2.0 ^b^	7.3 ^a^	0.570	<0.01
FAE, mU	4.2	4.5	4.5	0.47	0.85
AE, mU	21.6 ^b^	20.2 ^b^	30.2 ^a^	1.72	<0.01

^a,b^ Values in the same row with different small letter superscripts represent significant difference (*p <* 0.05), while with no letter or the same letter superscripts represent no significant difference (*p* > 0.05). CMCase, carboxymethyl cellulase; AVI, avicelase; XLY, xylanase; FAE, ferulic acid esterase; AE, acetyl esterase; SEM, mean standard error.

**Table 3:** The relative abundance of anaerobic fungal genera composition (belonging to the *Neocallimastigomycota* phylum) in donkey caecum and colon.

Items		Caecum	Ventral Colon	Dorsal Colon
*Cyllamyces*	Min	0.008	0.011	0.002
	Max	0.589	3.81	2.16
	Mean ± SD	0.21 ± 0.19	0.71 ± 0.52	0.69 ± 0.32
*Piromyces*	Min	0.002	0.002	0.009
	Max	0.346	1.21	3.34
	Mean ± SD	0.12 ± 0.11	0.42 ± 0.18	1.24 ± 1.05
*Neocallimastix*	Min	ND	0.003	ND
	Max	0.002	2.67	5.66
	Mean ± SD	0.002	0.68 ± 0.66	5.66
*Buwchfawromyces*	Min	0.006	0.003	ND
	Max	0.014	1.88	0.95
	Mean ± SD	0.01 ± 0.004	0.94 ± 0.93	0.95
Unclassified_f_*Neocallimastigaceae*	Min	0.002	0.013	0.049
	Max	0.066	4.78	0.838
	Mean ± SD	0.03 ± 0.02	0.83 ± 0.57	0.41 ± 0.13
Unclassified_c_*Neocallimastigomycetes*	Min	ND	0.002	0.003
	Max	ND	0.18	0.03
	Mean ± SD	ND	0.06 ± 0.04	0.02 ± 0.01

Min, minimum value; Max, maximum value; SD, standard error; ND, not detected.

## Data Availability

The data that support the findings of this study are available from the authors.
